# Unveiling the Biosynthetic Pathway for Short Mycolic Acids in Nontuberculous Mycobacteria: Mycobacterium smegmatis MSMEG_4301 and Its Ortholog Mycobacterium abscessus MAB_1915 Are Essential for the Synthesis of α′-Mycolic Acids

**DOI:** 10.1128/spectrum.01288-22

**Published:** 2022-07-07

**Authors:** Cecilia B. Di Capua, Juan M. Belardinelli, Hugo A. Carignano, María V. Buchieri, Cristian A. Suarez, Héctor R. Morbidoni

**Affiliations:** a Laboratorio de Microbiología Molecular, Facultad de Ciencias Médicas, Universidad Nacional de Rosario, Rosario, Argentina; Johns Hopkins University School of Medicine

**Keywords:** α′-mycolic acid biosynthesis, *Mycobacterium smegmatis*, *Mycobacterium abscessus*, antibiotic susceptibility, drug development

## Abstract

Mycolic acids, a hallmark of the genus Mycobacterium, are unique branched long-chain fatty acids produced by a complex biosynthetic pathway. Due to their essentiality and involvement in various aspects of mycobacterial pathogenesis, the synthesis of mycolic acids—and the identification of the enzymes involved—is a valuable target for drug development. Although most of the core pathway is comparable between species, subtle structure differences lead to different structures delineating the mycolic acid repertoire of tuberculous and some nontuberculous mycobacteria. We here report the characterization of an α′-mycolic acid-deficient Mycobacterium smegmatis mutant obtained by chemical mutagenesis. Whole-genome sequencing and bioinformatic analysis identified a premature stop codon in MSMEG_4301, encoding an acyl-CoA synthetase. Orthologs of MSMEG_4301 are present in all mycobacterial species containing α′-mycolic acids. Deletion of the Mycobacterium abscessus ortholog MAB_1915 abrogated synthesis of α′-mycolic acids; likewise, deletion of MSMEG_4301 in an otherwise wild-type M. smegmatis background also caused loss of these short mycolates.

**IMPORTANCE** Mycobacterium abscessus is a nontuberculous mycobacterium responsible for an increasing number of hard-to-treat infections due to the impervious nature of its cell envelope, a natural barrier to several antibiotics. Mycolic acids are key components of that envelope; thus, their synthesis is a valuable target for drug development. Our results identify the first enzyme involved in α′-mycolic acids, a short-chain member of mycolic acids, loss of which greatly affects growth of this opportunistic pathogen.

## INTRODUCTION

Although Mycobacterium tuberculosis and Mycobacterium bovis are two of the most important pathogens of the genus Mycobacterium, other mycobacterial species—known as nontuberculous mycobacteria (NTM)—such as M. avium, M. ulcerans, M. fortuitum, M. abscessus, M. chelonae, and the recently reported M. cosmeticum—are rapidly becoming a reason for concern due to their increasing role in human diseases and the difficulty in treating the infections (1–5). A common feature of all the members of the genus is their extremely low cell permeability, explained in part by a thick, complex cell wall structure (6, 7). One of the hallmarks of this structure is mycolic acids, high-molecular-weight, α-alkyl, β-hydroxy fatty acids with a general structure of [R1-CH(OH)-CH(R2)-COOH], essential components of the cell walls of mycobacteria (6). The mycolic acid structure contains fatty acids of extraordinary length (up to 80 C long) that bear a number of chain modifications, such as the presence of double bonds, cyclopropane, and keto, methoxy, and epoxy groups. The complexity of the pathway and the chemical characteristics of mycolic acids posed a formidable challenge for the study of their synthesis through lipid biochemistry. Importantly, the availability of the genome sequence of M. tuberculosis as well as of several NTM, including that of the saprophytic model organism Mycobacterium smegmatis—helped in identifying genes of the mycolic acid synthetic pathways (7, 8). Due to its essentiality, mycolic acid synthesis and mycolic acid transport are targets of drugs in clinical use, such as isoniazid and ethionamide, as well as of new molecules, inhibitors of the mycolic acid flippase, MmpL3 (9–11). Thus, the complete understanding of the mycolic acid biosynthetic pathway makes it possible to identify and fully exploit possible novel targets. This strategy has thoroughly been used in M. tuberculosis, leading to the identification of the majority of the enzymes linked to synthesis, transport, and deposition of these long-chain α-hydroxylated branched fatty acids into the cell wall structure (6, 7). Several NTM possess a slightly different repertoire of mycolic acids in terms of their final structure; examples of that are the wax ester mycolic acids present in Mycobacterium avium and the epoxy-mycolates present in Mycobacterium fortuitum and M. smegmatis. A more striking difference is that, in addition to α-mycolates, short-chain mycolates designated as α′-mycolates are present in pathogenic (Mycobacterium abscessus, Mycobacterium chelonae, Mycobacterium simiae, M. fortuitum) and nonpathogenic NTM species (M. smegmatis, Mycobacterium gilvum, Mycobacterium vaccae) (12, 13). The length of the meromycolate moiety of this short-chain family is C38 to C44, while its total length is C60 to C66, thus raising the question of its biosynthesis pathway compared to the longer (C72 to C81) α-mycolates. So far, no enzymes linked to the synthesis of α′-mycolates have been identified. Importantly, the presence of numerous paralogs in each species makes it a difficult task to identify genes encoding such enzymes by a bioinformatics approach. Alternative strategies have proven successful, as shown by the recent identification of HadD (MSMEG_0948)—carrying out a critical step leading to synthesis of both α- and epoxy-mycolic acids—by utilization of a two-hybrid approach (13). Thus, HadD is a potentially important target for the development of drugs specifically killing impervious NTM, which are usually resistant or poorly affected by standard antitubercular drugs. Using a simple strategy, we resorted to a genetic screen (previously used by Liu and Nikaido for the isolation of mycolic acid temperature-sensitive [TS] M. smegmatis mutants) based on a phenotype of hypersusceptibility to lipophilic drugs (HSD) due to cell envelope alterations (14). During this work we isolated several (~200) mutants with the general HSD-TS phenotype, five of which had alterations in fatty acid or mycolic acid biosynthesis. One such mutant was studied in detail and found to be defective in the synthesis of epoxy-mycolic acids (12), while a second one displayed remarkable alterations in the mycolic acid profile, colony morphology, and growth features identical to those described by Lefebvre et al. (13). We here describe in detail the characterization of a third mutant deficient in α′-mycolates, identifying MSMEG_4301 as the first reported gene leading to the synthesis of these short mycolates. This work also led to the identification of MAB_1915, deletion of which suppressed the synthesis of this mycolic acid family in M. abscessus. Moreover, expression in Mycobacterium bovis var. BCG strain Pasteur 1173 resulted in synthesis of α′-mycolates, strongly suggesting that the identified gene is the only one required for this synthesis. Future work will make it possible to design drugs against this potential target in NTM species that make this family of mycolic acids.

## RESULTS

### Isolation of mutants with a drug-hypersensitive, temperature-sensitive phenotype.

**FIG 1:**
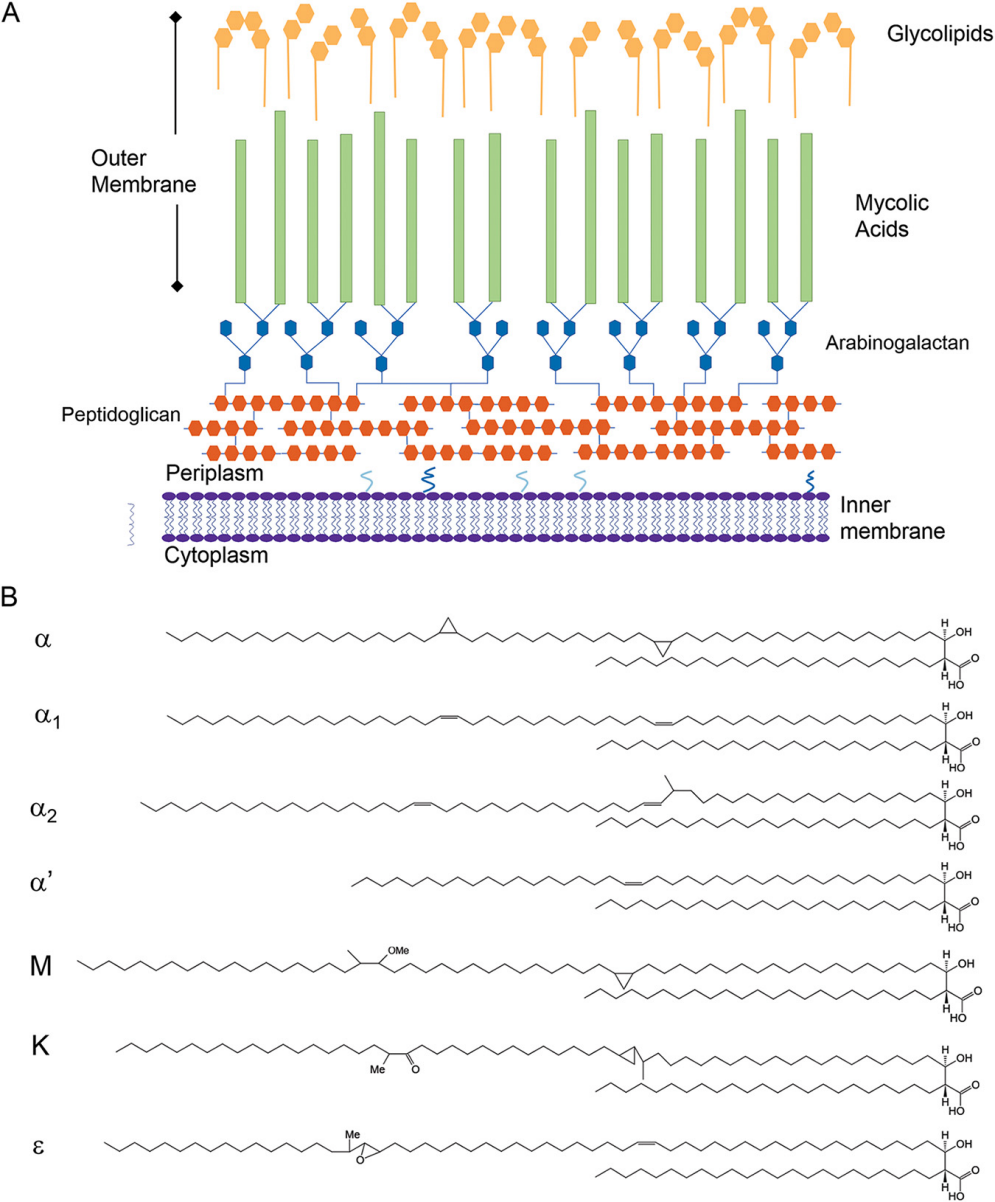
(A) Scheme of the mycobacterial cell envelope. The cytoplasmic membrane, peptidoglycan-arabinogalactan core, and an outer layer composed of mycolic acids and glycolipids are indicated. (B) Structure of the mycolic acids present in the mycobacterial cell envelope. The α- (78 to 80 carbons), α1- and α2- (78 and 79 carbons, respectively), and α′- (64 carbons) mycolates are nonoxygenated types; keto (K), methoxy (M), and epoxy (ε) mycolates are oxygenated mycolic acids.

**FIG 2:**
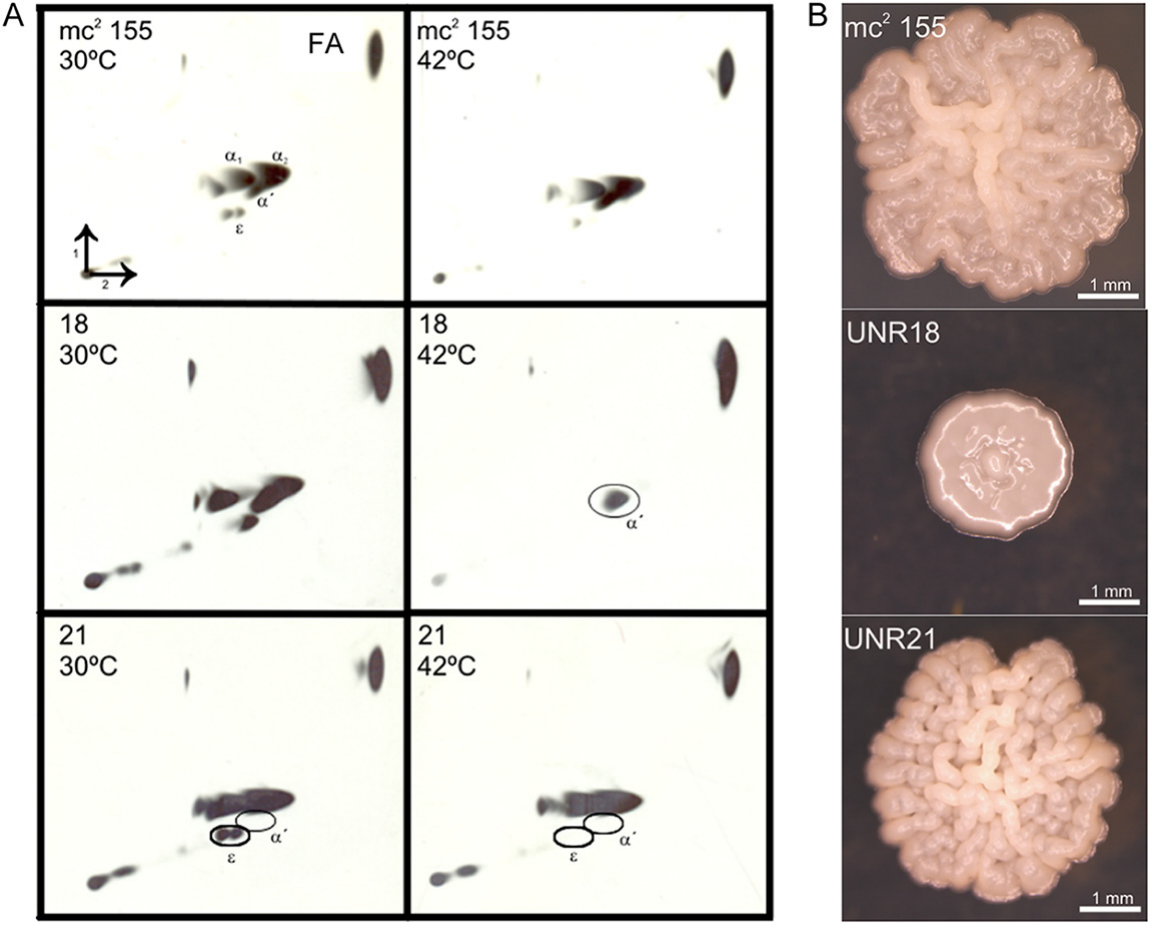
Temperature-dependent loss of specific mycolic acid families (A) and impact on colony morphology (B) in mutants derived from M. smegmatis mc^2^155. (A) Cultures of strain mc^2^155 (wild-type) and mutants UNR18 and UNR21 were grown at 30°C until the mid-log phase, divided in two aliquots (one kept at 30°C and one shifted to 42°C), and labeled by addition of 1-[^14^C]-acetate (1 μCi/mL). The cells were harvested, FAMEs and MAMEs were extracted, and aliquots (≈80,000 cpm) were analyzed by 2D-TLC. Hexane:ethyl acetate (95:5 vol/vol) was the solvent for the first dimension, while the second dimension was run three times in petroleum ether:diethyl ether (85:15 vol/vol). Detection of the radiolabeled FAMEs and MAMEs was done by autoradiography, exposing the TLC plates to X-ray films for 24 h at −70°C. α, α′, and ε correspond to α-mycolates, α′-mycolates, and epoxy-mycolates, respectively. FA, fatty acids. (B) Aliquots of fresh cultures of each strain grown at 30°C were plated on 7H9 Gly-ADS-Congo red (100 μg/mL) solid medium and incubated at 30°C for 5 days before growth was inspected under a binocular scope.

Members of the genus Mycobacterium have a very complex cell envelope composed of peptidoglycan, arabinogalactan and long-chain branched fatty acids—mycolic acids—along with other glycolipids and complex lipids of various structures depending on the species (6). The mycolic acids interact with other molecules of the outermost layers of the cell; thus, lack of or decreased amounts of one or more mycolic acid families would translate into changes in the entry of drugs into the cell cytoplasm, causing a drug-hypersensitive (HSD) phenotype. Since we considered that the potential of a previously described screening method based on drug hypersensitivity due to cell envelope alterations had not been fully exploited (14), we used it to isolate mutants deficient in mycolic acid biosynthesis using the saprophytic species M. smegmatis mc^2^155. This species has three types of mycolic acids, two nonoxygenated (the shorter α′- and the longer α-mycolic acids) and the oxygenated epoxy-mycolic acids (15) (Fig. 1). For this purpose, we performed chemical mutagenesis and analyzed roughly 8,700 mutagenized colonies grown at 30°C. Screening for mutants showing a hypersensitive phenotype to subinhibitory concentrations of novobiocin (50 μg mL^−1^) or crystal violet (2 μg mL^−1^) and subsequent analysis of their temperature sensitivity (TS) led to the isolation of five mutant clones, two of which (UNR18 and UNR21) were chosen to be studied in detail. Drug susceptibility testing showed that both mutants were hypersusceptible to the antibiotics used for selection as well as to rifampicin, tetracycline, and chloramphenicol, with UNR18 showing a higher susceptibility than UNR21 (Table 1). Interestingly, UNR18 displayed a severe change in colony morphology, yielding smooth, small glossy colonies, while UNR21 produced colonies of slightly smaller size than the parental strain at 30°C upon 4 to 5 days of incubation (Fig. 2A). To verify that the mutants were affected in the synthesis of mycolic acids at high temperature, we performed their analysis by metabolic labeling with the precursor 1[^14^C] acetic acid, extraction, and separation by bi-dimensional thin-layer chromatography (TLC). Our results showed that UNR18 exhibited defects in the contents of α- and epoxy-mycolates and UNR21 lacked α′- mycolates (Fig. 2B). Surprisingly, synthesis of α′-mycolates was abrogated at both low and high temperatures, suggesting a nonessential role for these mycolates. While this work was in process, HadD, a novel fatty acid synthase type II (FASII) enzyme intervening in the synthesis of α- and epoxy-mycolates, was described (13). The similarity of the phenotypes between our UNR18 TS mutant and a Δ*hadD* mutant (increased susceptibility to drugs, smooth colony morphology, and loss of α- and epoxy-mycolic acid families) led us to consider that UNR18 could be a mutant deficient in HadD activity, and thus we focused on UNR21.

**TABLE 1:** MIC values for M. smegmatis mutants hypersusceptible to hydrophobic drugs

M. smegmatis strain	MIC (μg/mL) for:					
	Rifampicin	Crystal violet	Novobiocin	Tetracycline	Chloramphenicol	Isoniazid
Wild type	64	16	128	0.5	16	5
UNR18	4	2	8	0.06	2	2.5
UNR21	16	4	16	0.25	8	2.5

### Whole-genome sequencing of UNR21 suggests a role for MSMEG_4301 in the synthesis of α′-mycolic acids in M. smegmatis.

In order to identify the mutation leading to the loss of α′-mycolic acids, we performed whole-genome sequencing of UNR21; to this end, chromosomal DNA was extracted from UNR21 and our lab stock of M. smegmatis mc^2^155 and sequenced. Bioinformatic analysis of the mutations present in UNR21 that were absent in both our wild-type strain and in the chromosomal sequence available in a public database (Mycobrowser, https://mycobrowser.epfl.ch) (16) revealed a large number of mutations (*n* = 1,520). This was an expected result because the mutant was generated by chemical mutagenesis. However, careful scrutiny of the mutated genes, discarding those that were unrelated to possible fatty acid synthesis pathways, allowed us to narrow the list to nine candidate genes with annotated functions compatible with that synthesis (Table 2). An in-depth analysis of these genes showed that five of them contained mutations leading to amino acid changes, three contained frameshifts, and an eighth candidate, MSMEG_4301, annotated as a fatty acyl CoA synthase, contained a mutation changing a Q97 to a stop codon (Table 2).

**TABLE 2:** Mutations detected in UNR21 and impact on the corresponding open reading frame (ORF)

Gene	Function	Mutation	Protein change
MSMEG_1821	Acyl-CoA dehydrogenase	g452a	R151K
MSMEG_1904	Acyl-CoA dehydrogenase	g768a	K256K
MSMEG_2029	3-Ketoacyl-ACP/CoA reductase	Insertion c31	Frameshift at AA11
MSMEG_2228	Short-chain dehydrogenase/reductase family	c169t	P57S
MSMEG_3392	Acyl-CoA dehydrogenase domain protein	Insertion c199, c220, c222	QYGGHGR replaced by SVRWARAA at AA67
MSMEG_3490	Acyltransferase (similar to Rv0517)	g929a	G310D
MSMEG_4301	Acyl-CoA synthase, Fatty acyl-AMP ligase (FAAL) FadD	c289a	Q97 to stop
MSMEG_4727	Pks 5, mycocerosic acid synthase	c2795t	S932F
MSMEG_6511	Acyl-CoA dehydrogenase	Insertion c1047	Frameshift at AA349

Inspection of the genomic context of MSMEG_4301 in M. smegmatis showed that this gene is followed downstream by MSMEG_4300, encoding a hypothetical transcriptional regulator of the AmtR family. The upstream gene, MSMEG_4302, encoding a hypothetical adenylate cyclase, is transcribed in the opposite direction of MSMEG_4301 (see Fig. S1 in the supplemental material).

### MSMEG_4301 orthologs are present in NTM species containing α′-mycolates.

On the basis of our findings, we next analyzed if genes related to MSMEG_4301 could be identified in NTM species such as M. abscessus, M. chelonae, and M. fortuitum that contain α′-mycolates (16). Analysis of the KEGG database using the sequence similarity database pinpointed orthologs of MSMEG_4301 in all the α′-mycolates containing NTM (17); a reassessment of the list of nine candidates showed that some of those were present in some but not all NTM species containing α′-mycolates, and some were present on mycobacterial species that do not synthesize those short mycolates (Table 3). Thus, the correlation between the presence of MSMEG_4301 orthologs and α′-mycolates reinforced our hypothesis of the role of this gene in the synthesis of α′-mycolates.

**TABLE 3:** Orthologs of MSMEG_4301 and other candidate genes in mycobacterial species

Gene	Function	Orthologs in other mycobacteria^*a*^
MSMEG_1821	Acyl-CoA dehydrogenase	M. marinum, **M. fortuitum**, M. ulcerans, M. avium, M. kansasii
MSMEG_1904	Acyl-CoA dehydrogenase	**M. goodii, M. fortuitum, Mycobacterium sp. strain VKM, Mycobacterium sp. strain JLS, Mycobacterium sp. strain KMS, Mycobacterium sp. strain MCS**, M. rhodesiae, **M. chubuense, M. vanbaalenii, M. vaccae, *M. gilvum***, M. phlei, M. neoaurum
MSMEG_2029	3-ketoacyl-ACP/CoA reductase	***M. goodii*, Mycobacterium sp. VKM, M. fortuitum**, M. dioxanotrophicus
MSMEG_2228	Putative beta-ketoacyl acyl carrier protein [ACP] reductase	**M. fortuitum, M. thermoresistibile, *M. phlei*, M. vaccae, M. vanbaalenii**, M. avium, M. paratuberculosis, M. intracellulare, ***M. chubuense*, *M. gilvum***
MSMEG_3392	Acyl-CoA dehydrogenase domain protein	***M. goodii*, *M. phlei*, *M. chubuense*, Mycobacterium sp. VKM, M. fortuitum**, M. chimaera, M. intracellulare, **M. vanbaalenii**, M. marseillense, ***M. gilvum***, M. sinense
MSMEG_3490	Acyltransferase (similar to Rv0517)	***M. goodii*, M. fortuitum**, M. canetti, M. tuberculosis, M. bovis
MSMEG_4301	Acyl-CoA synthase, fatty acyl-AMP ligase (FAAL) FadD	***M. goodii*, *M. thermoresistibile*, *M. chubuense*, M. litorale, M. fortuitum, *M. vanbaleenii*, M. vaccae, *M. gilvum*, M. abscessus, M. immunogenum, M. saopaulense, M. chelonae**
MSMEG_4727	Pks 5, mycocerosic acid synthase	***M. goodii*, *M. phlei***, *M. canetii*, M. avium, M. kansasii, M. marinum
MSMEG_6511	Acyl-CoA dehydrogenase	***M. goodii*, M. fortuitum, Mycobacterium sp. VKM, Mycobacterium sp. JLS, Mycobacterium sp. KMS, Mycobacterium sp. MCS, *M. chubuense*, M. vaccae, *M. gilvum*, M. vanbaalenii**, M. paratuberculosis, M. avium, M. terrae, M. chimerae

a Species with α′-mycolates are shown in bold.

**FIG 3:**
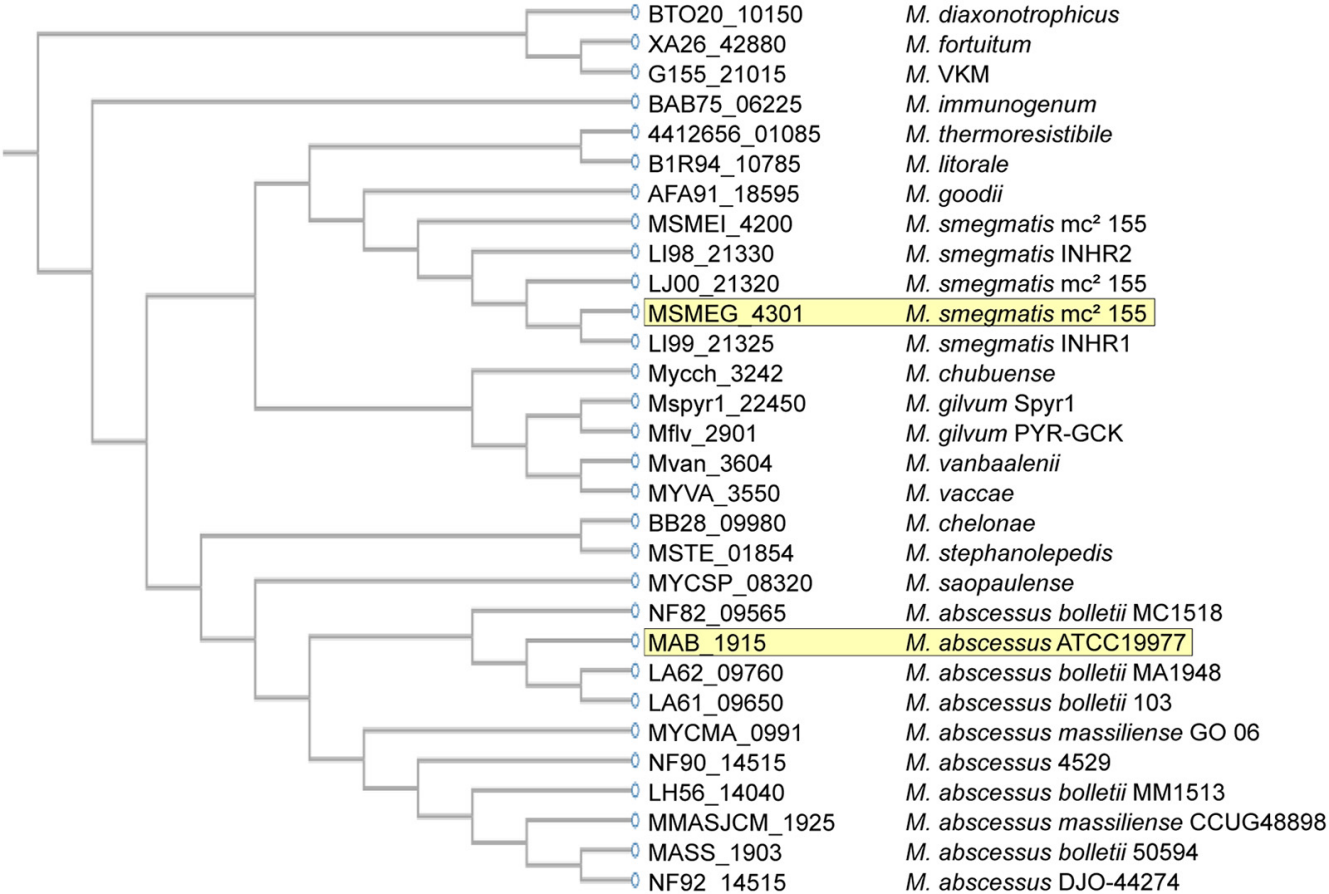
Dendrogram of MSMEG_4301 orthologs in α′-mycolate containing mycobacteria. Ortholog genes were identified in KEGG using the sequence similarity database (17).

Phylogenetic analysis showed that the orthologs differ in their relatedness, with those present in M. abscessus complex subspecies being more closely related to each other but clearly separated from that of M. smegmatis. Notably, the M. fortuitum ortholog is the most divergent one, raising questions about the time of the acquisition or intraspecies paralog evolution of this gene (Fig. 3).

### MSMEG_4301 is a putative fatty acyl AMP ligase.

**FIG 4:**
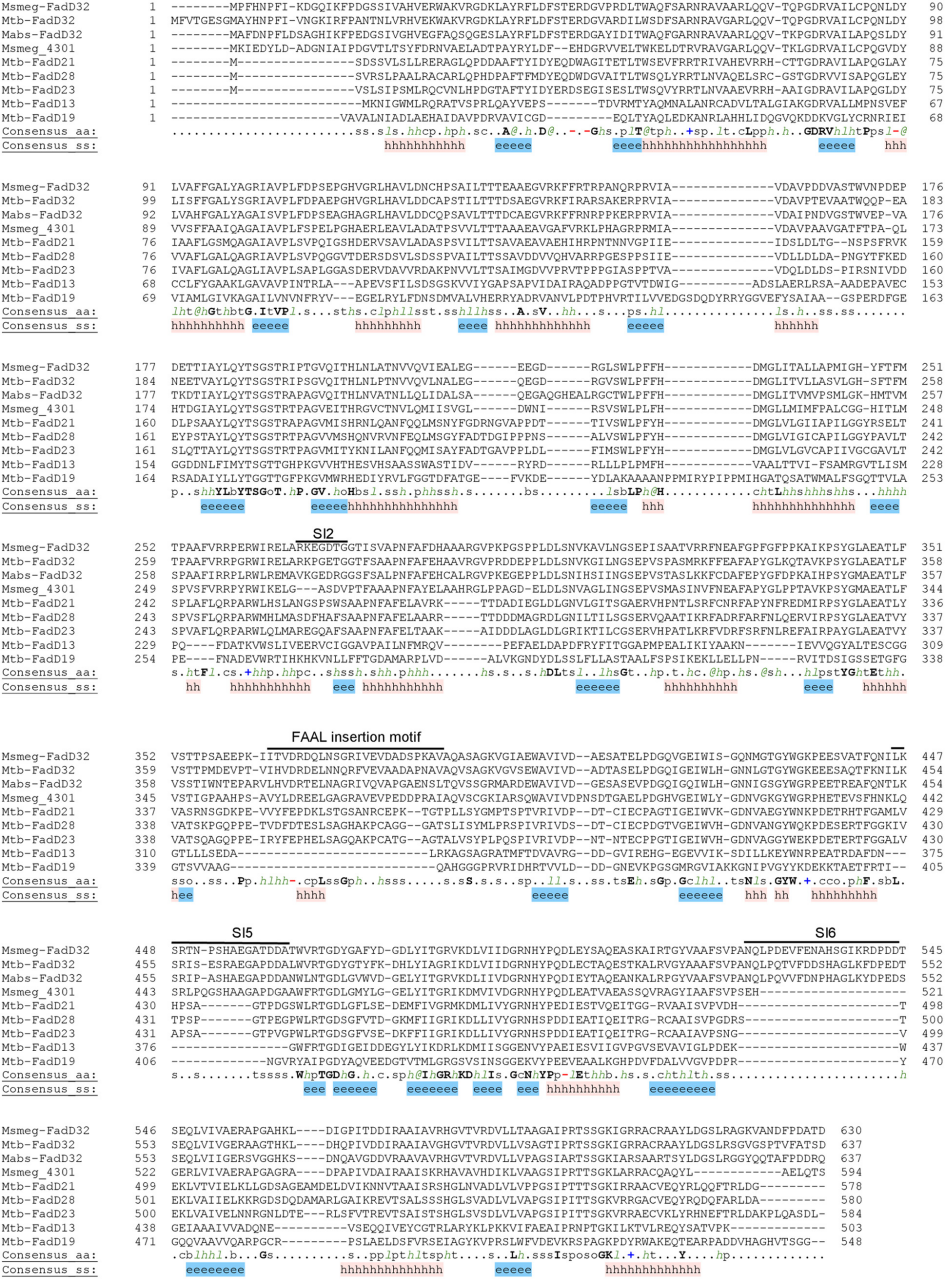
Structure-based alignment of FAAL and FACS enzymes. M. tuberculosis FadD32, M. smegmatis FadD32, and M. abscessus FadD32 were aligned against MSMEG_4301 and five other M. tuberculosis FadD enzymes, three of them belonging to the FAAL family (FadD21, FadD28, and FadD23) and two belonging to the FACS family (FadD13 and FadD19). The insertion sequence distinctive of FAAL enzymes as well as insertion sequences characteristic of FadD32 enzymes (SI2, SI5, and SI6) are highlighted.

In order to gain insight on the reaction catalyzed by MSMEG_4301, we compared it to 22 enzymes encoded by genes annotated as FAAS (fatty acyl ACP CoA synthetase) that are present in the M. tuberculosis genome having the general designation of *fadD*. The FadD family is heavily represented in mycobacterial genomes, consisting of 36 enzymes in M. tuberculosis; all of them are involved in fatty acid activation using ATP to produce acyl-adenylate intermediates (18). However, there are two types of enzymatic activity in this family, one is that of fatty acyl CoA synthetases (FACS) that catalyze a second reaction in which acyl chains are transferred to coenzyme A (CoA), while the second one is fatty acyl AMP ligases (FAAL), which transfer the activated acyl chains onto the acyl carrier protein (ACP) domains of their cognate polyketide synthase (Pks) (19). Importantly, the last condensation step between a C24 to C26 acyl chain and meromycolates is carried on by the concerted action of three enzymes: AccD4, FadD32 (Rv3801c), and Pks13. The genes coding these enzymes cluster together in the M. tuberculosis and M. smegmatis genomes (20). A characteristic feature of FAAL-type enzymes is a short insertion of 20 to 25 amino acids in length, sufficient to prevent the formation of acyl-CoA derivatives (21). Our analysis of MSMEG_4301, by comparing its amino acid sequence to that of M. smegmatis FadD32 and other well-known M. tuberculosis FACS and FAAL enzymes, revealed the presence of this insertion specific to the latter enzymes spanning from positions 336 to 366 in the protein (Fig. 4), suggesting that MSMEG_4301 belongs into the FAAL family.

Sequence alignment of M. smegmatis and M. tuberculosis FadD32 enzymes with MSMEG_4301 and its orthologs revealed a high degree of similarity between these enzymes, with only one major difference, which was the absence of a FadD32-specific insertion (designated SI6) in MSMEG_4301 and related enzymes (such as M. abscessus Mabs_1915) from α′-mycolate containing NTM (Fig. S2). Interestingly, a three-dimensional structure comparison between MAB_0179 (FadD32) and MAB_1915 shows that SI6 is present in the former but not in the latter, suggesting differences in their roles (Fig. S3).

### Deletion of M. abscessus MAB_1915 or MSMEG_4301 causes loss of α′-mycolates in these species.

**FIG 5:**
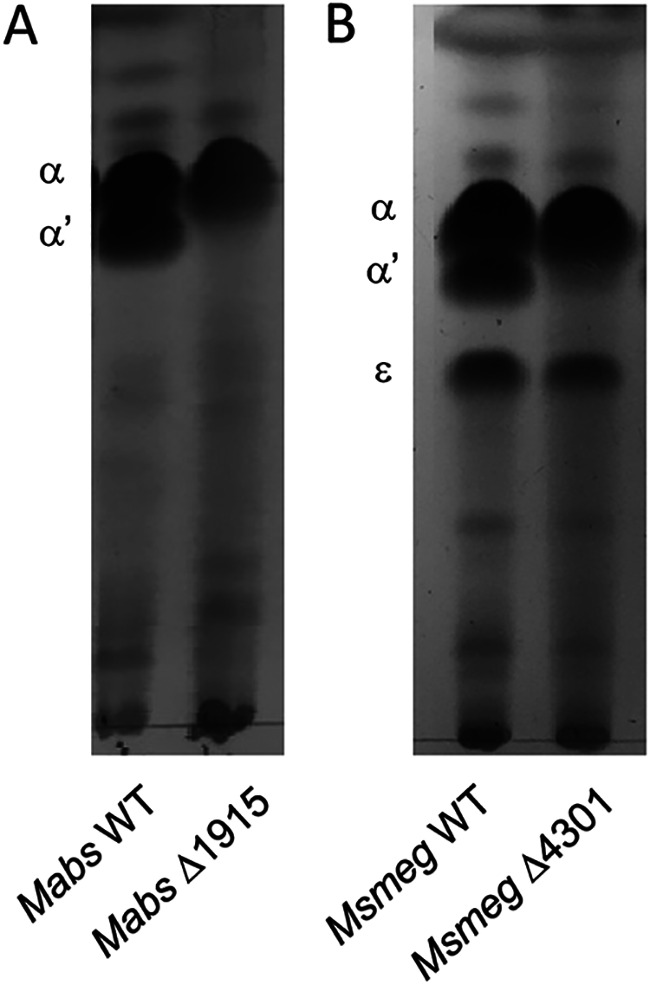
Deletion of MSMEG_4301 or MAB_1915 is sufficient for the loss of α′-mycolic acids in M. smegmatis and M. abscessus, respectively. MAMEs extracted from M. smegmatis wild type (WT), M. smegmatis ΔMSMEG_4301, M. abscessus WT and M. abscessus ΔMAB_1915 (generated from comparable amounts of cells) were spotted on TLC plates, developed three times in hexane:ethyl acetate (95:5 vol/vol), and revealed with CuSO_4_.

Infections due to NTM are becoming more common and are difficult to treat; among them, M. abscessus is a major opportunistic pathogen affecting particularly susceptible individuals with structural or functional lung conditions such as cystic fibrosis (CF), chronic obstructive pulmonary disease (COPD), and bronchiectasis (4). Due to the importance of this mycobacterial species, we deleted MAB_1915, the ortholog of gene MSMEG_4301, by using a suicide plasmid system. The mutant obtained displayed the expected loss of α′-mycolates (Fig. 5A), confirming that this gene is indeed involved in their synthesis. Of note, the analysis of other extractable lipids did not show appreciable differences except for an increase in trehalose monomycolates (TMM) and trehalose dimycolates (TDM), between parental and mutant strains (Fig. S4). The observed change in intensity in TDM and TMM is most likely due to the loss of TDM molecules containing either two α′-mycolate chains or one of each, α- and α′-mycolate; thus, only TDM molecules carrying two α-mycolates would be detected. Similarly, TMM molecules would contain only α-mycolates.

A recent combined effort to generate freely available mycobacterial resources generated 588 precise gene deletions using the “recombineering” methodology (22). One of the deleted genes was MSMEG_4301; upon requesting the strain (the generous gift of K. Derbyshire and J. Wade), we analyzed its mycolic acid contents, finding that, in agreement with our results on UNR21 and M. abscessus, the deletion eliminated the synthesis of α′-mycolates (Fig. 5B).

**FIG 6:**
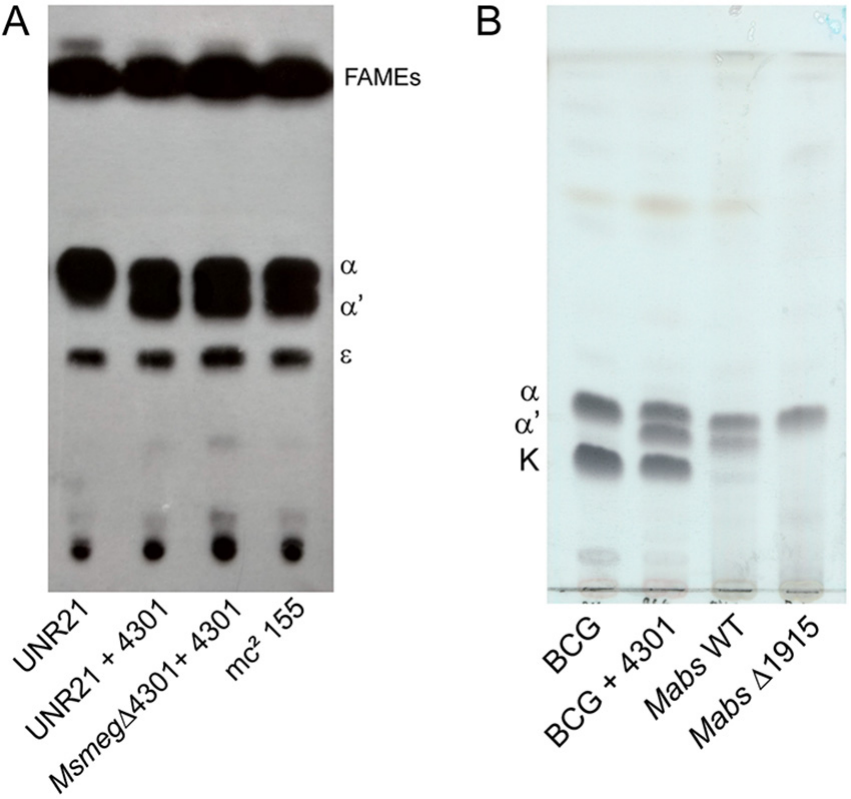
(A and B) Expression of MSMEG_4301 in UNR21 or M. smegmatis Δ4301 (A) and M. bovis BCG (B) is enough to allow α′-mycolate synthesis. Cultures of M. smegmatis mc^2^155, UNR21, UNR21:pMSMEG_4301, and M. smegmatis Δ4301:pMSMEG_4301 were labeled with 1-[^14^C]-acetate at 30°C, and their lipids were extracted. FAMEs and MAMEs were separated in TLC plates developed three times in hexane:ethyl acetate (95:5 vol/vol) and revealed by autoradiography. Electroporation of the same plasmid into M. bovis BCG Pasteur, extraction of nonradioactive lipids, and TLC analysis shown in panel B indicate production of α′-mycolates. M. abscessus is included as the control to show relative mobility of each mycolic acid.

In order to confirm the role of MSMEG_4301, we cloned it in an extrachromosomal plasmid and introduced the construct by electroporation into UNR21 or *Msmeg* Δ4301; the construct was able to restore full synthesis of α′-mycolates, thus confirming that the mutation leading to premature termination of the translation of this gene was the cause of the deficiencies in mycolate composition in the UNR21 mutant strain (Fig. 6A). Thus, we decided to name these enzymes FadD32S, in view of the fact that they are fatty acyl-AMP ligases involved in the synthesis of short α′-mycolic acids.

Moreover, to learn whether other gene products are required to produce α′-mycolates, the pMV261:: MSMEG_4301 plasmid was introduced by electroporation into M. bovis BCG strain Pasteur 1173—which lacks keto-mycolates due to an *mma*3 mutation (23) and thus only contains α- and keto-mycolates, making it simpler to detect a third mycolate species—leading to the synthesis of α′-mycolates in this slow-growing mycobacterium (Fig. 6B). This result strongly suggests that MSMEG_4301 and MAB_1915 are indeed the only genes necessary for its biosynthesis.

### Absence of α′-mycolates affects growth in M. abscessus but not in M. smegmatis.

Our initial studies using UNR21 showed that this mutant displayed a higher susceptibility to lipophilic compounds than the wild-type M. smegmatis strain; however, because of its generation by chemical mutagenesis, other mutations could be interfering with a clean analysis. Thus, we determined the MIC values for crystal violet, rifampicin, novobiocin, tetracycline, and chloramphenicol on M. smegmatis Δ4301; our results were identical to those obtained with UNR21, indicating that the loss of α′-mycolates was responsible for the increased susceptibility to these compounds (data not shown). Drug sensitivity testing of M. abscessus Δ1915 showed that the MIC values for several drugs were unchanged; however, the mutant strain was more susceptible to novobiocin, crystal violet, and rifampicin, compounds used on our isolation of UNR21 (Table 4).

**TABLE 4:** Drug susceptibility of M. abscessus WT and M. abscessus Δ1915^*a*^

	MIC (μg/mL) for:	
Drug	M. abscessus WT	M. abscessus Δ1915
CV	64	8
Novobiocin	>256	32
Streptomycin	>256	>256
Ciprofloxacin	8	8
Clofazimine	0.5	0.25
Azithromycin	16	16
Amikacin	32	32
Rifampicin	>256	64
Isoniazid	>256	>256
Tetracycline	>256	>256

a MICs were determined after 5 days of incubation.

**FIG 7:**
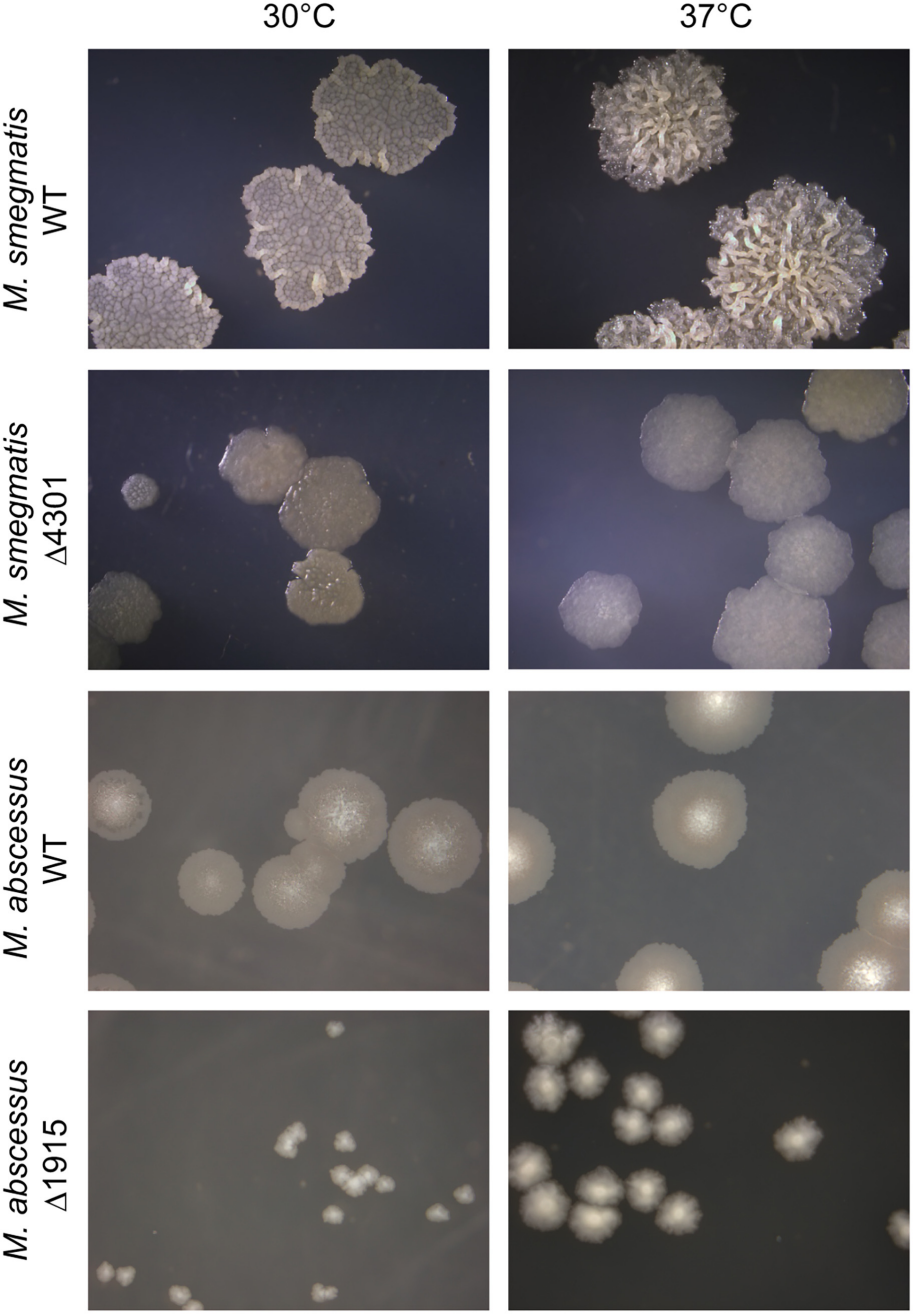
Loss of α′-mycolates affects colony size in M. abscessus. Dilutions of stationary cultures of both M. smegmatis and M. abscessus wild-type strains and their derived mutants were spotted on solid medium and incubated at either 30°C (5 days for M. smegmatis, 7 days for M. abscessus) or 37°C (3 days for M. smegmatis, 5 days for M. abscessus) followed by microscopy observation.

In order to address the impact of the loss of α′-mycolates on both species, we analyzed colony morphology and growth features and tested the mutants for their ability in biofilm formation, sliding motility, aggregation, and susceptibility to SDS. Interestingly, colonies of M. smegmatis Δ4301 and the wild-type strain were as similar in size at 30°C and 37°C, although the morphology of the colonies in the mutants showed a glossy and less organized appearance (Fig. 7). However, surprisingly, colony size was drastically affected in Mabs_Δ1915, yielding much smaller colonies at both temperatures (Fig. 7).

Biofilm formation, sliding motility, aggregation, and susceptibility to SDS did not show substantial differences between the wild type and mutants of both species (data not shown). Thus, the impact of the loss of α′-mycolates is heavier on the structure or functional performance of M. abscessus compared to that of M. smegmatis; further studies would be needed to understand the basis for these differences between species.

## DISCUSSION

Using chemical mutagenesis of M. smegmatis mc^2^155 and screening for increased susceptibility to lipophilic compounds—an approach successfully used by Liu and Nikaido (14)—we isolated several drug-hypersusceptible mutants. Analysis of their mycolic acids yielded three mutants lacking epoxy-mycolic acids, α- and epoxy-mycolic acids, and α′-mycolic acids, respectively. However, since the genetic defect in each one of the first two mutants had already been identified and reported elsewhere (12, 13), we concentrated our efforts on the third mutant, deficient in α′-mycolic acids. Upon genome sequencing and bioinformatics analysis, MSMEG_4301, encoding a hypothetical fatty acyl-CoA synthetase, was successfully identified as the gene carrying a mutation that created a premature stop codon. MSMEG_4301 and orthologs are exclusively present in mycobacterial species containing α′-mycolates; in support of our proposal of the role of MSMEG_4301, Segniliparus rotundus, a recently identified species containing α′-, α-, and the very long-chain α^+^-mycolic acids, has orthologs of both FadD32 and FadD32S (Srot_2768 and Srot_3008) with 72% and 55% similarity, respectively (24).

The function of MSMEG_4301 was confirmed by three different approaches: (i) deletion of MAB_1915 in M. abscessus ATCC 19977 and of MSMEG_4301 in M. smegmatis led to loss of α′-mycolic acids (Fig. 5); (ii) restoration of synthesis of α′-mycolic acids was obtained upon introduction of a normal copy of the gene (Fig. 6A); and (iii) c- α′-mycolic acid production was detected in M. bovis BCG after transformation with a clone carrying MSMEG_4301 (Fig. 6B).

Interestingly, although both deletion mutants shared an increased susceptibility to lipophilic drugs, the effect of the loss of α′-mycolic acids on colony morphology is very different in both species, being much subtler in M. smegmatis. In this species, able to grow in a very wide temperature range, the amount of epoxy- and α′-mycolates is modulated by temperature in an opposite way, with the former increasing their concentration at low temperatures and the latter, at high temperatures (12). In this regard, the recent identification in M. smegmatis of HadD, a dehydratase belonging to the mycobacterial FASII system, has shed some light on NTM mycolic acid biosynthesis. Deletion of the *hadD* gene in M. smegmatis led to the loss of both long-chain α- and epoxy-mycolic acids, while α′-mycolic acid remained unchanged and was the only mycolic acid present in the mutant. The M. smegmatis Δ*hadD* deletion mutant had increased sensitivity to rifampicin, SDS, and low temperature (13). However, although M. smegmatis Δ4301, devoid of α′-mycolic acids, also showed increased sensitivity to rifampicin and novobiocin, it did not show differences in susceptibility to high temperature, as should be expected.

Mycobacterial chromosomes are highly enriched in genes with possible functions in fatty acid synthesis or degradation. M. smegmatis is no exception to that, containing 22 genes annotated as acyl-CoA synthases. Moreover, mycobacteria contain a large number of complex lipids that are synthesized through the concerted action of specialized polyketide synthases and their cognate acyl-CoA synthases (20, 25–27). In this field, the study of the mechanisms of synthesis of mycolic acids and complex lipids such as phthiocerol dimycoserosate brought to light a novel mechanism of fatty acyl activation which uses AMP as acceptor, producing acyl adenylates instead of using coenzyme A (19, 28). It has been shown by Arora et al. that the major sequence difference between fatty acyl-CoA synthases (FACS) and fatty acyl AMP ligases (FAALs) is the presence in the latter group of a stretch of 20 to 25 amino acids that is inserted at position 362 (numbering varies for each enzyme); deletion of this stretch led to the change of the activity of the FAAL to that of an FACS (21). The presence of this insertion motif in MSMEG_4301 and MAB_1915 clearly indicates that these enzymes belong to the FAAL family. Thus, our results suggest these FAALs activate shorter monounsaturated meromycolic acids (C38 to C44) to yield α′-mycolic acids, while FadD32 acts on the longer meromycolic acids (C52 to C60) found in the α and epoxy families. Whether the meromycolic acid activated by MSMEG_4301/MAB_1915 is also loaded onto Pks13 or to another Pks is not currently known. It is common for FAAL and its cognate Pks to be on the same genomic cluster; the facts that there are no genes encoding Pks enzymes in the vicinity of either MSMEG_4301 or MAB_1915 (Fig. S1) and that expression of MSMEG_4301 was sufficient for synthesis of α′-mycolates in M. bovis BCG strain P1173 (Fig. 6B) suggests that Pks13 may also be responsible for the condensation step leading to the shorter α′-mycolic acids.

Structure-based alignment of M. smegmatis and M. tuberculosis FadD32 enzymes with MSMEG_4301 and orthologs revealed a high degree of similarity between these enzymes at the structural level. The only major difference was the absence of a FadD32-specific insertion (designated SI6) in MSMEG_4301 and orthologs (Fig. S3) (29). SI6 has a short α-helix and is located at the surface of the enzyme, far from the substrate binding site; however, it is not clear how it could determine the length of the meromycolic acid substrate.

**FIG 8:**
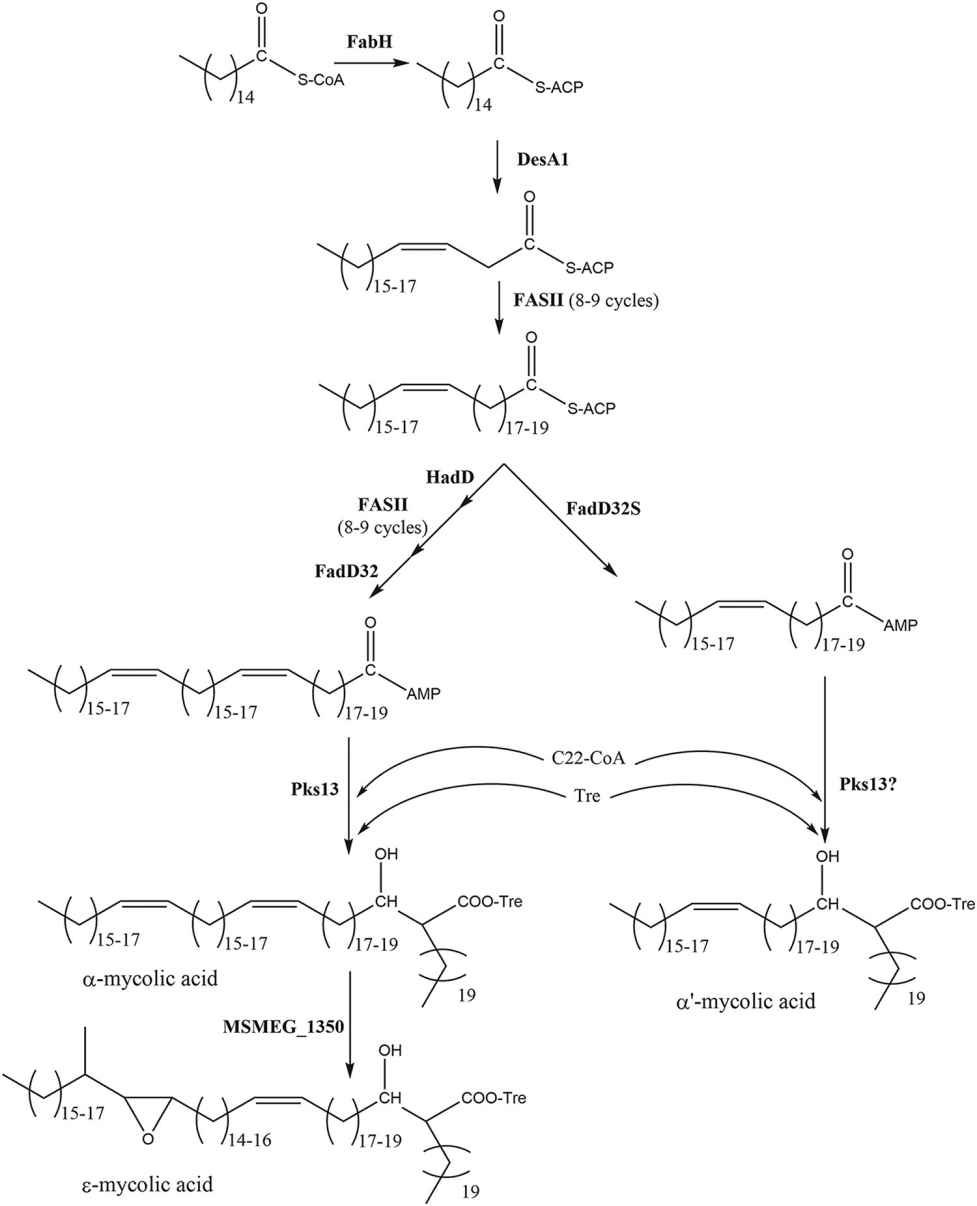
Proposed model of synthesis of mycolic acids in M. smegmatis and other mycobacteria containing α′-mycolates. After introduction of the distal *cis* double bond in the fatty acyl chains, the meromycolate is extended through a series of FASII elongation cycles up to a C36 to C40 chain length. At this point, the short meromycolate can either be activated by FadD32S and condensed via Pks13 with a C24 fatty acyl-CoA to form the short C60-C64 α′-mycolic acid or be further dehydrated by HadD and extended through a series of FASII cycles to produce a C56 to C60 α-meromycolate. This meromycolate is activated by FadD32 and transferred to Pks13, generating the α-mycolic acid esters. Finally, in M. smegmatis, α-mycolic acids are synthesized by the action of MSMEG_1350 on the α-mycolic acid.

The evidence presented here supports the biosynthetic model shown in Fig. 8 for M. smegmatis and other α′-mycolic acid-containing species. After the introduction of the first unsaturation (distal), the meromycolic acid is extended by FASII up to C38 to C44. At this point, the meromycolic acid is either activated by FadD32S or further desaturated by HadD. In the first case, the activated fatty acyl chain is loaded onto Pks13, which catalyzes the addition of the C22 side chain to give a mature α′-mycolic acid. In contrast, after HadD adds a second unsaturation (proximal), the chain is further extended by FASII and then activated by FadD32 and loaded onto Pks13 to synthesize the longer α- and epoxy-mycolic acids.

A puzzling aspect derived from our results is the evolutionary preservation of the pathway for α′-mycolates, as it seems to be dispensable in M. smegmatis but important for normal growth in M. abscessus (Fig. 7). Interestingly, α′-mycolates are present in the opportunistic pathogens M. fortuitum, M. abscessus, and M. chelonae, responsible for increasing numbers of very hard-to-treat infections in humans. These species are intrinsically resistant to several antibacterial drugs and also show diminished susceptibility to antitubercular drugs (3); thus, the identification of novel targets specific for NTM, such as HadD and FadD32S, is a highly desirable goal. Interestingly, MAB_1915 was recently identified in a screening of M. abscessus transposon mutants with decreased growth in macrophages, suggesting that absence of α′-mycolates affects macrophage survival in this species; our results showing a major alteration in colony size support these observations (30).

Since we have demonstrated that loss of the short α′-mycolates causes an increased susceptibility to drugs, and considering that HadD impairs synthesis of the longer-chain mycolic acids, further studies of these two enzymes and their interactions are warranted with the goal of developing novel drugs active on NTM species.

## MATERIALS AND METHODS

### Strains, culture media, and growth conditions.

M. smegmatis mc^2^155 and M. abscessus ATCC 19977—used as parental strains—and all the mutants derived from them were routinely grown in Middlebrook 7H9 broth (Difco) supplemented with 0.5% (wt/vol) glycerol and 10% (vol/vol) either ADS (albumin-dextrose-NaCl) or OADS (oleic acid-albumin-dextrose-NaCl). The same medium with the addition of 1.5% (wt/vol) agar (Difco) or Middlebrook 7H11 was used as solid medium. To avoid clumping of liquid cultures, Tween 80 (Sigma; 0.05 to 0.5% [vol/vol] for M. smegmatis and 0.05% [vol/vol] for M. abscessus) was used. For the sake of simplicity these media will be referred to as 7H9-Gly-ADS/(OADS)-Tw. Plates were incubated at 24, 30, 37, or 42°C for 10, 5 to 6, or 3 days with daily visual inspection. When M. bovis BCG strain Pasteur 1173 was used, liquid cultures were grown for 7 days in 7H9-Gly-ADS-0.05% Tw or 21 to 30 days in solid medium as described above.

### Chemical mutagenesis and mutant screening.

Cultures of M. smegmatis mc^2^155 were grown in 7H9-ADS-glycerol-0.5% (vol/vol) Tween until an optical density at 600 nm (OD_600_) of 0.8 was reached. At that point, 1 M NaOH was added to a final pH of 8.5, and 1-methyl-3-nitro-1-nitrosoguanidine (MNNG; Sigma-Aldrich) was added to a final concentration of 100 μg mL^−1^. The cultures were incubated with shaking at 30°C for 20 min and then centrifuged, and the supernatant was discarded. The cell pellets were resuspended on fresh medium and further incubated for 4 h at 30°C to allow for cell recovery. Aliquots of the mutagenized cultures were plated on 7H11-Gly-OADS and incubated at 30°C for 5 days until colonies were clearly visible. At this point the colonies were transferred to 7H11-Gly-OADS master plates and grown at 30°C for 5 days.

The desired mutants were selected based on a drug hypersensitivity phenotype as described by Liu and Nikaido (14). Briefly, colonies were replicated onto 7H11-ADS plates in the presence of novobiocin (50 μg mL^−1^) or crystal violet (2 μg mL^−1^), followed by incubation at 30°C for 5 days. Colonies displaying an increased sensitivity to novobiocin or crystal violet—judged by restricted growth or no growth in the presence of those compounds—were replicated onto 7H9-Gly-ADS plates and incubated at 42°C to identify temperature sensitive (TS) variants.

### Drug susceptibility testing.

MIC values of the parental and mutant strains were determined by plating ~1,000 CFU on 7H9-Gly-ADS agar plates in the presence of increasing concentrations of the drugs tested. The plates were incubated at 30°C for 5 days. MIC_99_ was defined as the minimum concentration of drug required to produce a 99% growth inhibition compared to the control condition (plate with no drug added).

### Phenotypic characterization of M. smegmatis and M. abscessus mutants.

Colony morphology was recorded by plating dilutions of freshly grown stationary-phase cultures of wild-type and mutant strains on 7H9-Gly-ADS agar plates followed by incubation at 30°C for 3 to 5 days and optical microscopy examination at low magnification (×10). When necessary, Congo red (100 μg/mL) was added to the plates to improve the visualization of colony morphology alterations.

Temperature and detergent sensitivity testing were performed on solid medium. To that end, liquid cultures grown in 7H9-Gly-ADS-0.05% (wt/vol) Tween 80, were adjusted to the same OD and serially diluted. Then, 5-μL aliquots of each dilution were spotted onto Middlebrook 7H10 medium, and plates were incubated at 24°C, 30°C, 37°C, and 42°C for 10 days, 7 days, 5 days, and 3 days, respectively, in the case of M. smegmatis strains. When required, SDS (0.05 or 0.5% [wt/vol] depending on the experiment) was added to the medium, and plates were incubated for 3 days at 30°C.

In order to determine the extent of aggregation of wild-type strains and their derived mutants, each strain was grown to the early stationary phase in 7H9-ADS-Gly, followed by spontaneous sedimentation (1 × *g*) for 10 min to separate aggregates. Afterward, the supernatants were carefully transferred to new tubes, and their OD_600_ was measured. The cell aggregates in the sediments were briefly agitated by vortexing with 4-mm glass beads before determining their OD_600_. The aggregation index (AI) was calculated as the OD_600_ supernatant/OD_600_ sediment ratio. Controls were made by using cultures grown in 7H9-ADS-Gly-0.5% Tw to reduce aggregation. In each case the aggregation assay was performed in triplicate in three independent experiments.

With the aim of studying whether the sliding motility was affected in the mutant strains, 10 μL of a stationary-phase culture of each mutant strain was spotted onto 7H9-0.3% (wt/vol) agar with no carbon source added; plates were sealed with Parafilm and incubated at 30°C for 7 days, followed by visual inspection and measurement of the diameter of the halo of visible growth.

Biofilm production by the wild-type strain and mutant strains was determined by adding 2 μL of each saturated culture in 12-well microtiter plates loaded with 4 mL of M63 medium supplemented with dextrose (2% wt/vol), Casamino Acids (0.5% wt/vol), MgSO_4_ (1 mM), and CaCl_2_ (0.7 mM). Alternatively, Sauton medium was used. Plates were sealed and incubated at 30°C for 5 days, with daily visual observation.

### Incorporation of 1-[^14^C] acetate into fatty and mycolic acids.

The parental and mutant strains were grown at permissive temperature in 7H9-Gly-ADS-Tw broth until an OD_600_ of 0.6, at which point each culture was divided into two aliquots and 1-[^14^C] acetate (sodium salt; 58.9 millicurie (mCi)/mmol; Perkin Elmer, Boston, MA) was added (1 μCi mL^−1^; 37 kiloBecquerel (kBq) mL^−1^) to each of them, followed by further incubation for 3 h at 30°C or 42°C. The resulting ^14^C-labeled cells were harvested by centrifugation at 5,000 × *g*, washed twice with distilled water, and kept frozen until use.

### Analysis of fatty and mycolic acids.

The extraction and analysis of *in vivo* radiolabeled fatty acids and mycolic acids was performed as previously described (12). In brief, ^14^C-labeled control and treated cells were subjected to alkaline hydrolysis in 15% (wt/vol) tetrabutylammonium hydroxide (TBAH; Fluka) at 105°C overnight, followed by the addition of 2 mL of CH_2_Cl_2_ and 100 μL of CH_3_I. The entire reaction mixture was then mixed on a rotator for 1 h and centrifuged, and the upper aqueous phase was discarded. The lower organic phase was then washed with water and dried at 55°C under nitrogen stream. The resulting pellet was extracted with ethyl ether and dried again before adding a small volume of CH_2_Cl_2_. A known aliquot (50,000 cpm) of the resultant mixture of fatty acid methyl esters (FAMEs) and mycolic acid methyl esters (MAMEs) from each strain under analysis was subjected to analytical one-dimensional thin-layer chromatography (TLC) using silica gel plates (5735 silica gel 60 F254; Merck) and hexane/ethyl acetate (95:5 vol/vol, three developments) as the solvent system.

For two-dimensional silver ion argentation TLC (2D-TLC), aliquots (~80,000 cpm) of the mixture of FAMEs and MAMEs were applied to silica gel plates previously impregnated with 10% (wt/vol) AgNO_3_ (up to 80% of the plate length). The plates were developed in the first direction with hexane/ethyl acetate (90:10 vol/vol) three times and in the second direction twice with petroleum ether/diethyl ether (85:15 vol/vol). Autoradiograms were obtained after exposure to Kodak BioMax XAR film at −70°C for 24 h.

When nonradioactive mycolic acids were studied, the extraction protocol was as described above except that the radioactive precursor was not added to cultures. Mono and bi-dimensional TLC were developed as described, and the different mycolic acid species present were visualized after spraying the plate with a solution of 10% (wt/vol) CuSO_4_ in 8% phosphoric acid followed by charring (31).

Total extractable lipids were obtained and analyzed as described elsewhere (32). The different lipid fractions were developed in chloroform:methanol:water (20:4:0.5 vol/vol) [A], 90:10:1 vol/vol [B], or 60:30:6 vol/vol [C]. For TAG and DAG (D) lipids were developed in hexane:diethyl ether:acetic acid (70:30:1 vol/vol). In all cases lipids were detected as described above (32).

### DNA extraction and bioinformatics analysis of genomic sequences.

Chromosomal DNA extraction of M. smegmatis mc^2^155 and its derived mutant UNR21 was done as described previously (33). Library construction and sequencing were performed according to Ioerger et al. using an Illumina HiSeq 2500 and 51 × 51-bp paired-end reads (33). UNR21 chromosomal stock and lab stock of the parental strain sequence alignment to the M. smegmatis mc^2^155 reference sequence (GenBank accession version number NC_018289.1) was carried out using Bowtie 2 (34). Variants including single nucleotide polymorphisms (SNPs) or insertions and deletions (indels), were identified with variant calling in Ugene (http://ugene.net). Mutations present in the lab stock parental strain and in UNR21 but absent in the M. smegmatis mc^2^155 reference sequence were disregarded. The sequences of the possible candidate genes mutated in UNR21 were visually inspected, and the impact of SNPs or indels was determined using EMBOSS Transeq (https://www.ebi.ac.uk/Tools/st/emboss_transeq/).

### Disruption of M. abscessus MAB_1915.

The M. abscessus ATCC 19977 MAB_1915 gene was disrupted by using an in-house-developed method based on the one previously described by Viljoen et al. (35). For this purpose, we cloned the *xylE* gene into pEM7/Zeo (Thermo Fisher) to get the suicide plasmid pZEXY as the backbone for deletions; then a 500-bp PCR product of an internal sequence of the MAB_1915 gene with primers 1915-Fw (TTTGGCGGCCGCCATCCGCAGCGTCAGCTGGCT) and 1915-Rv (GACGGCTAGCGCCCACTGACTGCGCGCGATCTT) was amplified, gel purified, digested with NheI and NotI, and cloned into pZEXY digested with the same enzymes. The resulting plasmid was electroporated into M. abscessus ATCC 19977, and transformants were selected on 7H11 ADS plus Zeocin (Invitrogen) 100 μg/mL. Colonies were checked with 1% catechol to identify single crossover recombinants in which the plasmid had recombined into the chromosome. Insertion on MAB_1915 was confirmed by PCR.

### Cloning of MSMEG_4301.

M. smegmatis MSMEG_4301 was amplified by PCR with primers 4301-Fw (GGTACATATGAAGATCGAGGATTACCTCGATG) and 4301-Rv (GGCCAAGCTTGGTCGTCATGCCCGGATCAGGA); the product was digested with NdeI and HindIII and ligated into pMV261 digested with the same enzymes to express the gene under the control of the *hsp60* promoter. M. smegmatis wild type and mutant UNR21 and M. bovis BCG var. Pasteur were transformed as described elsewhere (12) and selected on solid medium containing kanamycin (20 μg/mL).
