# Supplementary material for: Unveiling the Biosynthetic Pathway for Short Mycolic Acids in Nontuberculous Mycobacteria: Mycobacterium smegmatis MSMEG_4301 and Its Ortholog Mycobacterium abscessus MAB_1915 Are Essential for the Synthesis of α′-Mycolic Acids
Source: Microbiol Spectr. 2022 Jul 7;10(4):e01288-22. doi: 10.1128/spectrum.01288-22 (PMC9431677; doi:10.1128/spectrum.01288-22)
Supplement: Supplemental file 1 — Fig. S1 to S4. Download spectrum.01288-22-s0001.pdf, PDF file, 0.6 MB [file spectrum.01288-22-s0001.pdf]

Legends to Supplementary Figures

Figure S1: **Genomic region of *MSMEG_4301* and *MAB_1915*.**

Fig S2: Sequence alignment of FadD32S enzymes. Aminoacid sequences of FadD32S from *M. smegmatis*, *M. abscessus*, *M. thermoresistibile*, *M. chelonae*, *M. vanbalenii*, *M. vaccae* and *M. gilvum* against *M. smegmatis* and FadD32 of *M. tuberculosis* were aligned. Sequence insertions (SI2, SI5 and SI6) characteristic of FadD32 enzymes are highlighted. Also shown is the FAAL insertion motif.

Figure S3: **3D Structure of *Mabs* FadD32 and *Mabs* FadD32S.** Three-dimensional model of *M. abscessus* FadD32 (left) and FadD32S (right) obtained from MabeLLINI (*Mycobacterium ABscessus* modELLing INItiative, http://www.mabellinidb.science) (1) showing the site of the SI6 stretch missing from FadD32S enzymes.

Figure S4: TLC analysis of extracted lipids from *M. abscessus* ATCC 19977 WT and Δ1915. Total extracted lipids were developed in chloroform: methanol: water 20:4:0.5 (A), 90:10:1 (B) or 60:30:6 (C). For TAG and DAG (D) lipids ere developed in hexane: diethyl ether: acetic acid (70:30:1). TDM: trehalose dimycolates, TMM: trehalose monomycolates, PE: phosphatidylethanolamine, CL: cardiolipin, GPLs: glycopeptidolipids, PIMs: phosphatidylinositol mannosides, TAG: triacylglycerol and DAT: diacylglycerol.

Figure S1

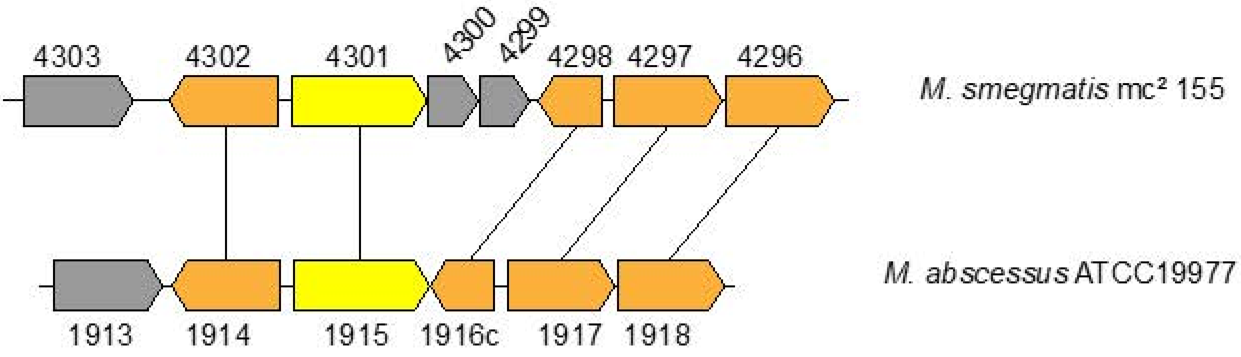

4303
4302
4301
4300
4299
4298
4297
4296
M. smegmatis mc² 155
M. abscessus ATCC19977
1913
1914
1915
1916c
1917
1918

Figure S2

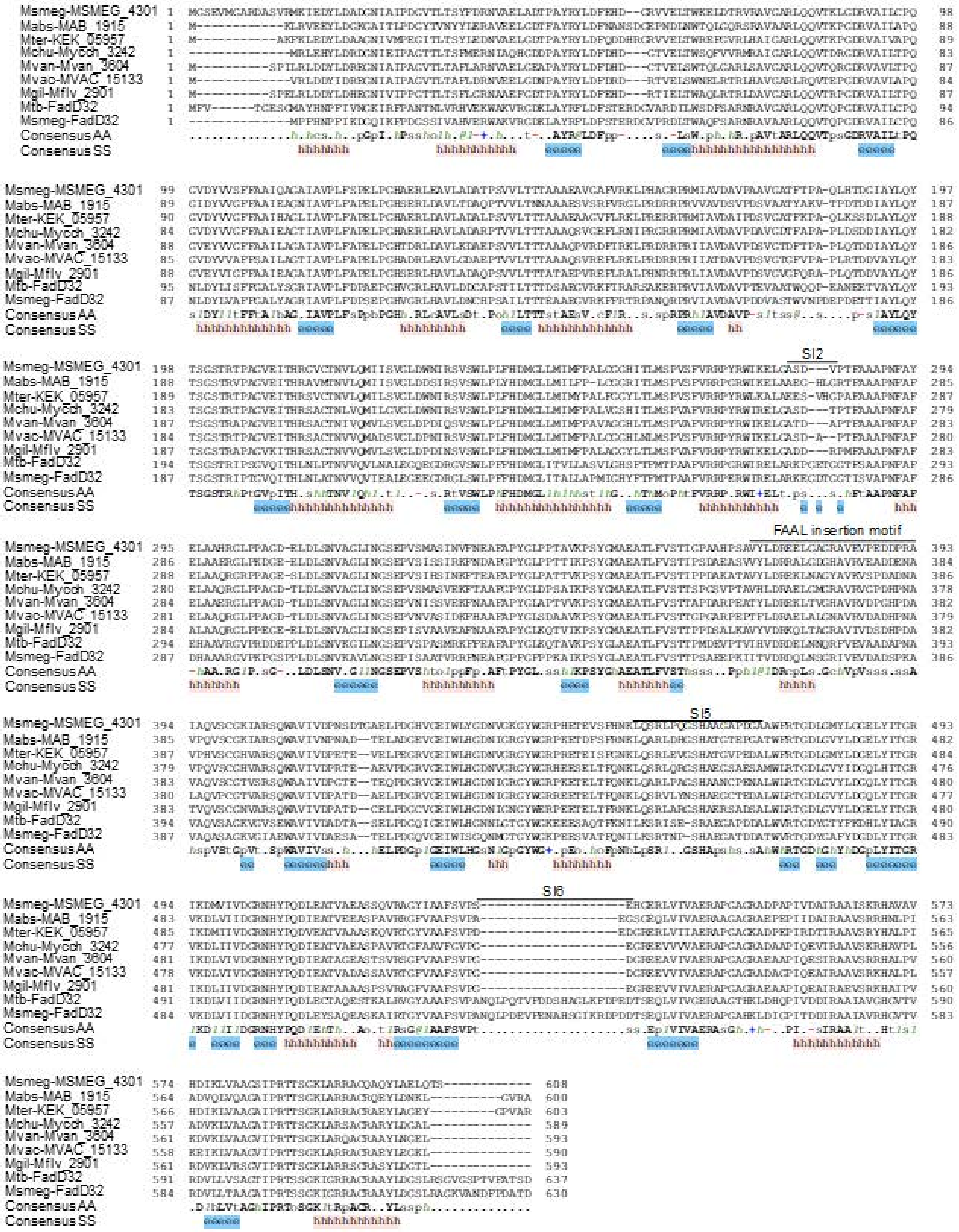
Msmeg-MSMEG_4301
Mabs-MAB_1915
Mter-KEK_05957
Mchu-Mycch_3242
Mvan-Mvan_3604
Mvac-MVAC_15133
Mgil-Mflv_2901
Mtb-FadD32
Msmeg-FadD32
Consensus AA
Consensus SS
SI2
FAAL insertion motif
SI5
SI6

Figure S4

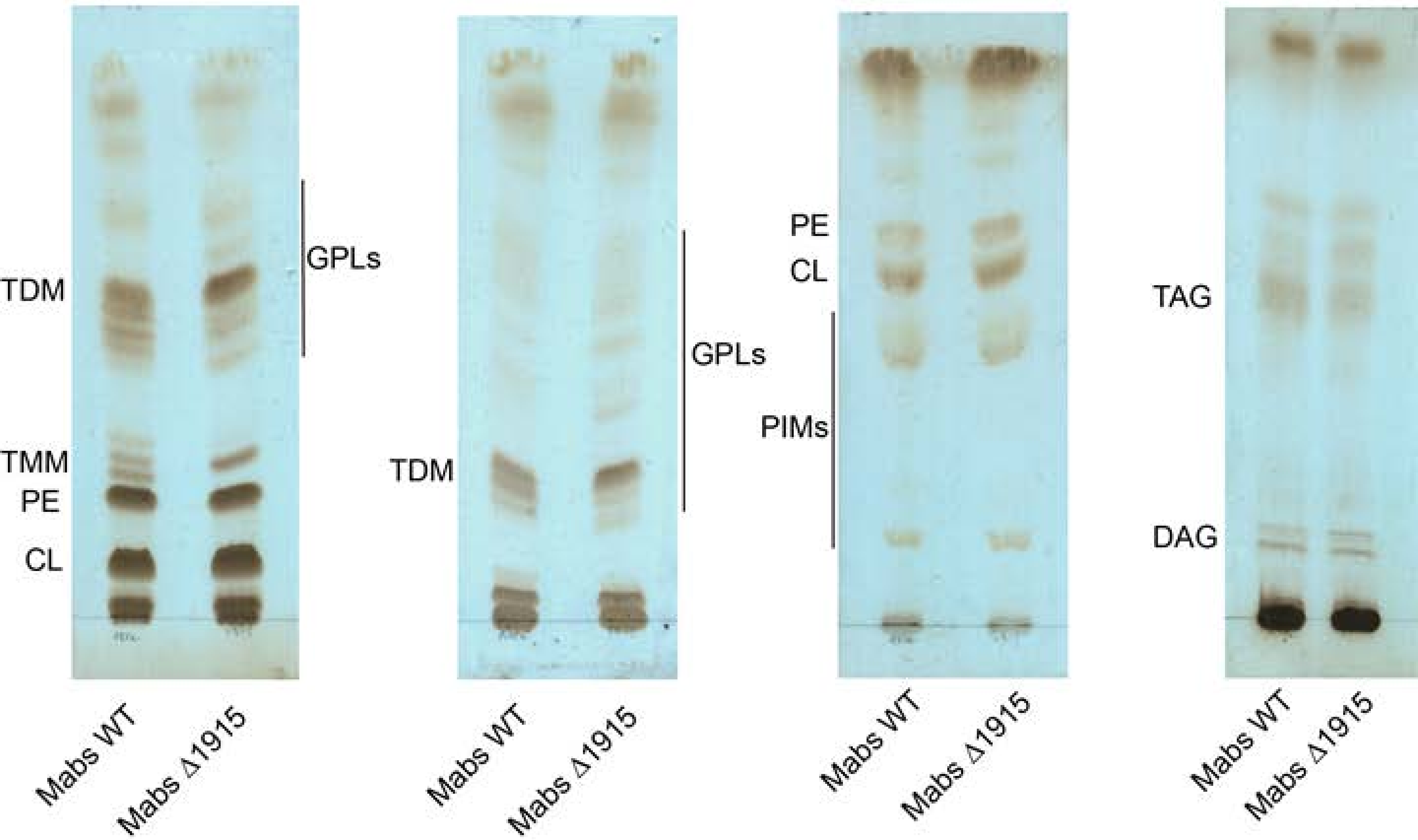
GPLs
TDM
TMM
PE
CL
Mabs WT
Mabs Δ1915
GPLs
TDM
Mabs WT
Mabs Δ1915
PE
CL
PIMs
Mabs WT
Mabs Δ1915
TAG
DAG
Mabs WT
Mabs Δ1915

Figure S3

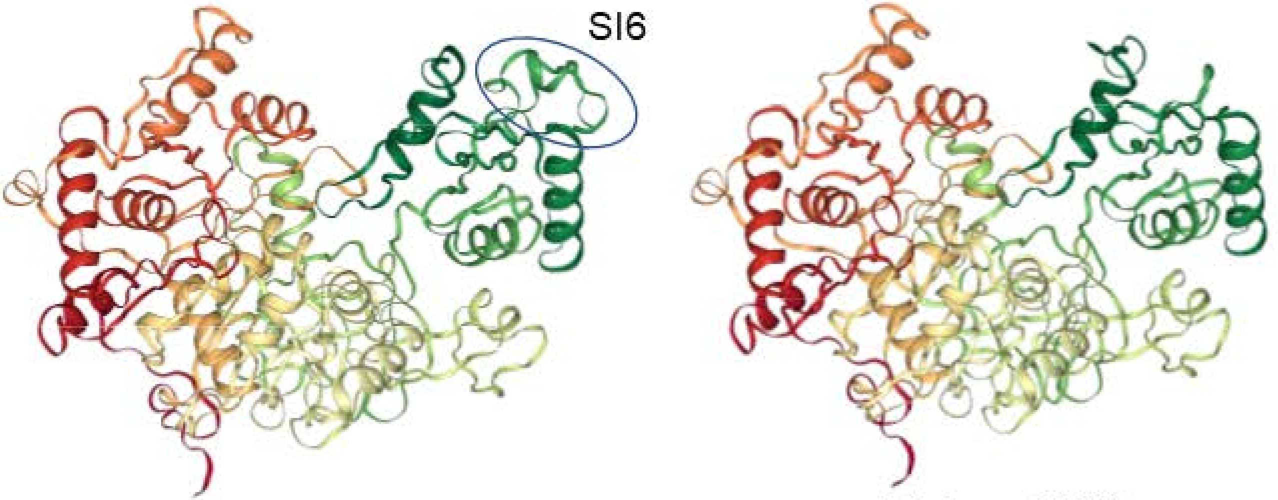

Mabs_0179(FadD32)

Mabs_4301

REFERENCES

1. Skwark MJ, Torres PHM, Copoiu L, Bannerman B, Floto RA, Blundell TL. 2019. Mabellini: a genome-wide database for understanding the structural proteome and evaluating prospective antimicrobial targets of the emerging pathogen Mycobacterium abscessus. Database (Oxford) 2019: baz113. https://doi.org/10.1093/database/baz113.
